# Exploring why patients with cancer consult GPs: a 1-year data extraction

**DOI:** 10.3399/bjgpopen19X101663

**Published:** 2019-10-02

**Authors:** Heidi Lidal Fidjeland, Ingvild Vistad, Svein Gjelstad, Mette Brekke

**Affiliations:** 1 Doctoral Research Fellow, General Practice Research Unit (AFE), Department of General Practice, Institute of Health and Society, University of Oslo, Oslo, Norway; 2 Doctoral Research Fellow, Department of Obstetrics and Gynecology, Sørlandet Hospital Kristiansand, Kristiansand, Norway; 3 Consultant, Department of Obstetrics and Gynecology, Sørlandet Hospital Kristiansand, Kristiansand, Norway; 4 Associate Professor, General Practice Research Unit (AFE), Department of General Practice, Institute of Health and Society, University of Oslo, Oslo, Norway; 5 Professor, General Practice Research Unit (AFE), Department of General Practice, Institute of Health and Society, University of Oslo, Oslo, Norway

**Keywords:** patients with cancer, general practitioners, follow-up, electronic health records, data extraction, neoplasms, primary health care

## Abstract

**Background:**

Survival rates of patients with cancer are increasing owing to improvements in diagnostics and therapies. The traditional hospital-based follow-up model faces challenges because of the consequent increasing workload, and it has been suggested that selected patients with cancer could be followed up by GPs.

The hypothesis of the study was that, regardless of the hospital-based follow-up care, GPs see their patients with cancer both for cancer-related problems as well as for other reasons. Thus, a formalised follow-up by GPs would not mean too large a change in GPs’ workloads.

**Aim:**

To explore to what extent patients with cancer consult their GPs, and for what reasons.

**Design & setting:**

A 1-year explorative study was undertaken, based on data from 91 Norwegian GPs from 2016–2017.

**Method:**

The data were electronically extracted from GPs' electronic medical records (EMR).

**Results:**

Data were collected from 91 GPs. There were 11 074 consultations in total, generated by 1932 patients with cancer. The mean consultation rate was higher among the patients with cancer compared with Norwegian patients in general. In one-third of the consultations, cancer was the main diagnosis. Apart from cancer, cardiovascular and musculoskeletal diagnoses were common. Patients with cancer who had multiple diagnoses or psychological diagnoses did not consult their GP significantly more often than patients with cancer without such comorbidity.

**Conclusion:**

This study confirms that patients with cancer consult their GP more often than other patients, both for cancer-related reasons and for various comorbidities. A formalised follow-up by GPs would probably be feasible, and GPs should prepare for this responsibility.

## How this fits in

Survival rates of patients with cancer are increasing owing to improvements in diagnostics and therapies. The traditional hospital-based follow-up model faces challenges because of the consequent increasing workload, and it has been suggested that selected patients with cancer could be followed up by GPs. This study confirms that patients with cancer already consult their GP more often than other patients do and that they consult for cancer-related reasons as well as for comorbidities. A formalised follow-up by GPs for selected patients should be discussed.

## Introduction

Improvements in diagnostics and therapies mean survival rates of patients with cancer are increasing. Furthermore, the proportion of the population that is older is increasing. Consequently, a growing number of patients with cancer need follow-up care. In addition to detecting recurrence and monitoring side effects of the disease and its treatment, there will be a growing demand for handling comorbid conditions and maintaining general health in the older cancer population. GPs have important roles along the cancer continuum, from cancer prevention and screening tests to delivery of palliative care. Nevertheless, there is a lack of formalised follow-up by GPs after cancer treatment, and follow-up care is mainly hospital-based. This traditional follow-up model faces challenges owing to increasing workload, and it has been suggested that selected patients with cancer could be followed up by GPs.^1–4^ GPs have reported broad experience in providing follow-up care to patients with cancer,^2,5^ and some randomised studies indicate that hospital-based follow-up provides no advantages compared with follow-up in primary care in terms of detection of recurrence or quality of life.^1,6,7^

Furthermore, when compared with the general population, studies among patients with colorectal, prostate, and breast cancer have shown that they have higher primary healthcare use during the first years following the diagnosis of cancer.^3,4,8,9^ A Danish study among 127 000 patients with cancer showed that they had 43–73% more GP consultations compared with the reference population 12 months after their diagnosis.^10^

It was hypothesised that, in addition to regular follow-up visits in hospitals, patients with cancer seek their GPs both for cancer-related problems and for other reasons.

The purpose of this study was to explore if Norwegian patients with cancer consult their GPs more often than the reference population, and for what purposes. In addition, the authors were interested in characteristics of GPs with more versus less contact with patients with cancer.

## Method

An explorative study was conducted based on 1-year data extracted from GPs' EMR in 2016–2017.

### Data collection

An electronic invitation to participate in the study was sent to GPs in all parts of Norway using email addresses obtained from the Norwegian Health Network (further information available from the authors on request). Some GPs were not connected to Norwegian Health Network and, thus, the email addresses were unknown. Other GPs were listed with the email address for their practice or for the municipal administration, and not their personal email address. Thus, the accuracy of the number of actual recipients could not be controlled.

A total of 191 GPs returned a consent email together with information about their sex, age, years in practice, and type of EMR system used (further information available from the authors on request). Thereafter, the responders received a new email with a code to anonymise their data together with a data extraction tool (further information available from the authors on request). Several responding GPs reported to have difficulties with their computer systems accepting the data extraction tool. This was especially a problem in municipal networks with advanced firewalls, and these GPs were excluded from participating. Owing to the technical problems, 91 responders were ultimately included in the analyses.

When the included GPs installed the data extraction tool into their EMR system and activated it by using the code, anonymous data were extracted and sent to a secure database at the University of Oslo. The electronic tool was developed by the firm Mediata AS based on the authors' specifications.

Retrospective data were retrieved from each GP's EMR on all patients registered with a cancer diagnosis in at least one consultation in the previous year. The following data were extracted: a list of each consultation by date during the previous year, sex, age, cancer diagnosis, and other diagnoses reported in the consultations, coded by the ICPC-2 (International Classification of Primary Care —Second Edition) system. According to the guidelines for GP accounting, the main diagnosis is written first.

The responding GP's patient list length was also retrieved from the Norwegian GP register.^11^

### Statistics

Data were analysed using SPSS Statistics (version 25). Simple descriptive quantitative statistical methods were used. Categorical data were expressed as frequencies and percentages, while continuous data were expressed as medians. Bivariate correlation between variables was analysed using Pearson correlation test. A *P*-value of <0.05 was considered to be statistically significant.

## Results

One-year data regarding patients with cancer was collected from 91 GPs. There were 11 074 consultations in total, generated by 1932 patients with cancer.

### Characteristics of the GPs

Demographic data for the GPs are provided in Table 1. According to data from the Norwegian Directorate of Health,^12^ the included GPs were comparable with Norwegian GPs in terms of sex distribution. Regarding age, the study had a lower proportion of GPs in the age group 30–39 years (19% versus 28%) and a greater proportion of GPs in the age group 55–66 years (38% versus 28%) compared with Norwegian GPs in general. Geographically, GPs from 17 out of 18 Norwegian counties were represented, including both rural and urban districts.

**Table 1.** Demographic characteristics of participating GPs, all Norwegian GPs, and included patients with cancer

	Participating GPs, *n* (%)	Norwegian GPs, *n* (%)	Patients with cancer, *n* (%)
	*n* = 91	*n* = 4759	*n* = 1932
**Sex**			
Female	29 (32)	1999 (42)	913 (47)
Male	59 (65)	2760 (58)	1017 (53)
Unknown	3 (3)	0 (0)	2 (0.1)
**Median age** **,** **years (range)**	50 (27–68)	47	69 (1–100)
<30 years	2 (2)	95 (2)	—
30–39 years	17 (19)	1333 (28)	—
40–54 years	32 (36)	1808 (38)	—
55–66 years	33 (38)	1333 (28)	—
>67 years	4 (5)	190 (4)	—
**Median years in practice (range)**	19 (1–41)	—^a^	—
****Median** number of consultations with** **patients** **with cancer (range)**	96 (0–456)	—^a^	—
****Median** number of** **patients** **with cancer** **consulting (range)**	19 (0–68)	—^a^	—
****Median** GP** **’** **s patient list size** **(range)**	1100 (430–1800)	—^a^	—
****Median** consultations per** **patient** **with cancer** **(range)**	—	—^a^	5 (1–40)

^a^Data not available.

The participating GPs differed widely in experience (1–41 years in practice), practice list size (430–1800 patients; mean list size in Norway is 1100 patients), number of included patients with cancer (0–68), and number of consultations with patients with cancer (0–456) (Table 1). There was significant correlation between the practice list size and the number of patients with cancer (*P* = 0.002) as well as between the practice list size and the number of consultations with patients with cancer (*P* = 0.001).

The study aimed to compare the GPs with fewer versus more patients with cancer, and the authors chose to compare GPs consulted by fewer than 10 patients with cancer (*n* = 19) with those consulted by more than 30 patients with cancer (*n* = 22). Those consulted by fewer than 10 patients with cancer in the past year had a median age of 43 years, 53% were male, and the median time in practice was 12 years. The median number of consultations with patients with cancer was 27. GPs consulted by more than 30 patients with cancer had a median age of 62 years, 86% were male, and their median time in practice was 29 years. The median number of consultations with patients with cancer for these GPs was 222 (data not shown).

### Characteristics of patients with cancer

**Figure 1.**
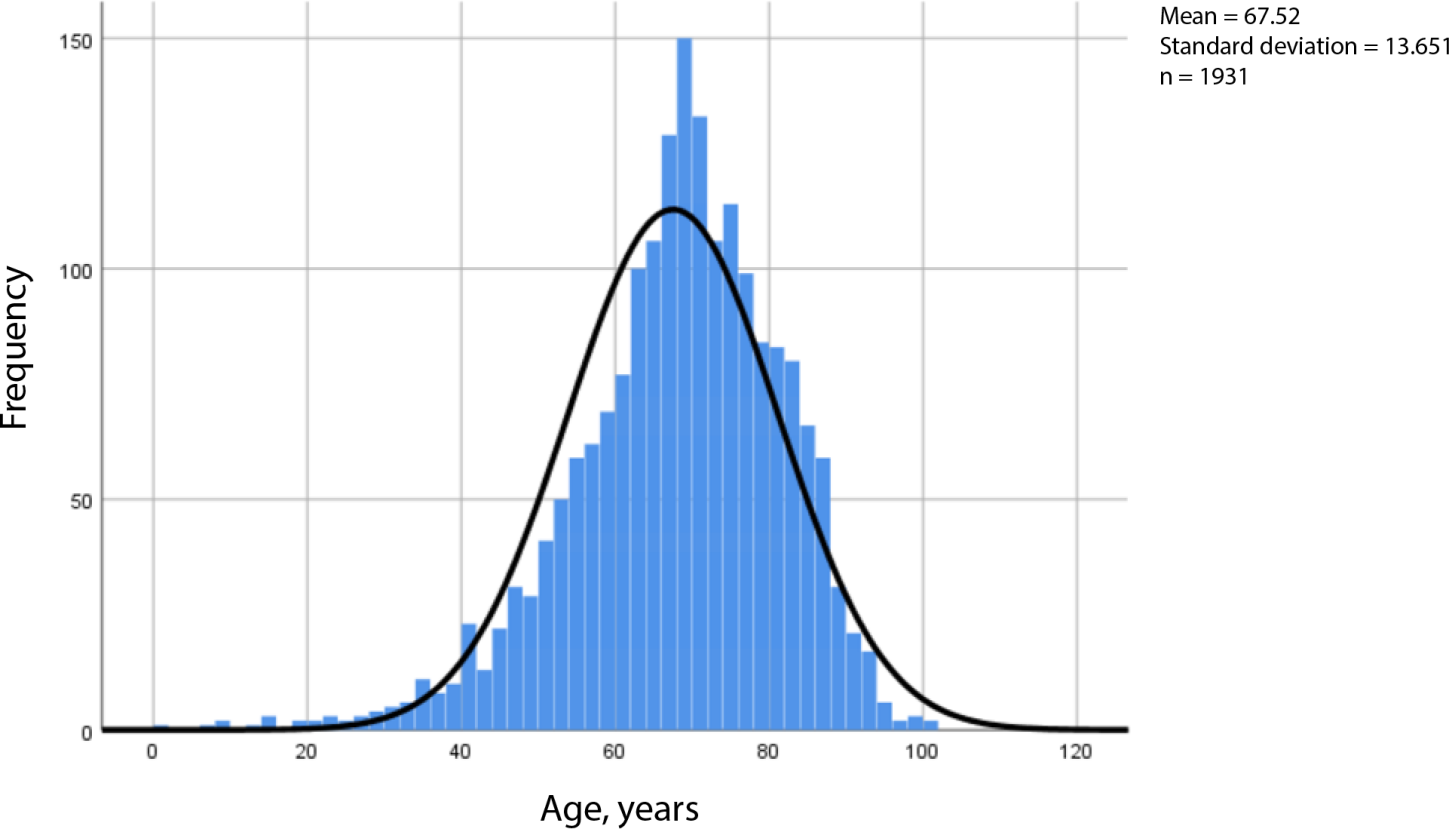
Consultations by age of patient with cancer in a 1-year study. Consultations: *n* = 11 074. Patients with cancer: *n* = 193. Sample: *n* = 1931 (missing data: *n* = 1)

Out of the 1932 cancer patients, 47% were women (Table 1). The median number of consultations per patient was five, varying from 1–40. The number of consultations related to patient's age is shown in Figure 1.

There was no significant difference in median age or the sex distribution between those who had more than five visits compared with those with fewer visits (Table 2), nor did they differ in having a psychological diagnosis or in the number of diagnoses per consultation.

**Table 2.** Characteristics of patients with cancer by frequency of visits to the GP in a 1-year study (consultations *n* = 11 074, patients with cancer *n* = 1932, GPs *n* = 91)

	Patients with cancer visiting <5 times per year, *n* (%)	Patients with cancer visiting ≥5 times per year, *n* (%)
	*n* = 2496 consultations	*n* = 8578 consultations
Sex^a^		
Female	460 (49)	453 (46)
Male	485 (51)	532 (54)
Median age, years	67	70
Consultations with two diagnoses	1123 (45)	3689 (43)
Consultations with three diagnoses	424 (17)	1458 (17)
Consultations with four diagnoses	125 (5)	600 (7)
P-diagnosis^b^ as first diagnosis in the consultation	75 (3)	343 (4)
P-diagnosis as second diagnosis in the consultation	50 (2)	257 (3)
P-diagnosis as third diagnosis in the consultation	25 (1)	86 (1)

^a^Missing data: *n* = 2. ^b^P-diagnosis = psychological diagnoses according to ICPC-2 (International Classification of Primary Care — Second Edition)

When the distribution of cancer diagnoses in the study was compared with the incidence of cancer in Norway 2013–2017 (Table 3)^13^ the distribution was relatively similar for the main cancer groups.

**Table 3.** Distribution of cancer diagnoses in 1932 patients consulting their GP during a 1-year study compared with the incidence of cancer in Norway 2013–2017^13^

**Cancer diagnosis**	**Frequency in study sample,** ***n* (%**)	**Incidence in Norway 2013–2017,** ***n* (%**)
**Total number**	1932	164 491
Prostate	446 (23)	27 081 (17)
Breast	336 (17)	17 079 (10)
Colorectal	249 (13)	22 003 (13)
Skin	241 (13)	20 179 (12)
Respiratory organs	105 (5)	16 467 (10)
Lymphoid and/or haematopoietic tissue	182 (9)	14 068 (9)
Stomach	17 (1)	2354 (1)
Pancreatic	15 (1)	3999 (2)
Gastrointestinal, not specified	59 (3)	—^a^
Gynaecological	70 (4)	8721 (5)
Urinary, bladder included	50 (3)	8435 (5)
Malignant tumour, not specified	43 (2)	—^a^
Kidney	41 (2)	4242 (3)
Central nervous system	24 (1)	5173 (3)
Musculoskeletal	22 (1)	—^a^
Male genital	18 (1)	1844 (1)
Thyroid	13 (1)	1973 (1)
Unknown	1 (0.05)	—^a^

^a^These data are not presented in the Cancer Registry of Norway.

### Diagnoses leading to consultations with the GP

Out of 11 074 consultations, 184 consultations lacked a diagnosis. A cancer diagnosis was the primary diagnosis in 33% of the consultations, and the only diagnosis in 17% (*n* = 1882) of the consultations. The distribution of the first, second, and third diagnosis given in the consultations is shown by code groups in Table 4.

**Table 4.** Distribution of diagnoses by ICPC-2 groups in 11 074 GP consultations with 1932 patients with cancer during 1 year

	Diagnosis 1	Diagnosis 2	Diagnosis 3
CODES	*n* (%)	*n* (%)	*n* (%)
Cancer code:	3653 (33)	1212 (25)	384 (21)
Other diagnoses by ICPC-2 code:			
A (General and unspecified)	814 (7)	403 (8)	182 (10)
B (Blood and/or immunity)	126 (1)	60 (1)	32 (2)
D (Digestive)	407 (4)	194 (4)	73 (4)
F (Eye)	151 (1)	46 (1)	15 (0.8)
H (Ear)	140 (1)	50 (1)	26 (1)
K (Cardiovascular)	1440 (13)	752 (16)	296 (16)
L (Musculosceletal)	1155 (10)	512 (11)	187 (10)
N (Neurological)	168 (2)	140 (3)	55 (3)
P (Psychological)	415 (4)	264 (6)	104 (6)
R (Respiratory)	675 (6)	240 (5)	100 (5)
S (Skin)	632 (6)	188 (4)	72 (4)
T (Endocrin and/or metabolic)	509 (5)	410 (9)	198 (11)
U (Urological)	379 (3)	191 (4)	72 (4)
W (Family planning)	18 (0.2)	8 (0.2)	2 (0.1)
X (Female genital)	69 (0.6)	17 (0.4)	14 (0.8)
Y (Male genital)	124 (1)	50 (1)	29 (2)
Z (Social problems)	10 (0.1)	16 (0.3)	2 (0.1)
Process codes	5 (0.05)	17 (0.4)	14 (0.8)
Missing diagnoses	184 (2)	2 (0.08)	
Total	11 074 (100)	4772 (100)	1857 (100)

Diagnosis 1 = main diagnosis set by the GP. Diagnosis 2 = second diagnosis. Diagnosis 3 = third diagnosis. ICPC-2 = International Classification of Primary Care — Second Edition

The primary diagnoses were distributed across all diagnostic code groups. Apart from the cancer codes, the most frequent diagnoses were placed within the K (cardiovascular [13%]) and the L (musculoskeletal [10%]) group. There were fewest diagnoses within the groups Z (social problems [0.1%]), W (family planning [0.2%]), and X (female genital [0.6%]).

Within the cardiovascular group, the most frequently used diagnoses were K86 (hypertension uncomplicated [29%]) and K78 (atrial fibrillation [22%]). Among musculoskeletal diseases, the diagnoses were more evenly distributed and L87 (bursitis or tendinitis or synovitis) was the most frequent (8%). Out of the 4% who had psychological primary diagnoses, P76 (depressive disorder) was the most frequent (21%).

## Discussion

### Summary

The 91 GPs participating in the study were consulted by 1932 patients with cancer during 1 year, and the median number of consultations per patient was five (mean 5.7). In one-third of the consultations, cancer was the main diagnosis. Apart from cancer, cardiovascular and musculoskeletal diagnoses were most common. Patients with cancer with several diagnoses or psychological diagnoses did not consult their GP significantly more often than patients with cancer without such comorbidity.

The GPs with most contact with patients with cancer were older and more experienced than those with less contact with patients with cancer.

### Strengths and limitations

Data retrieval directly from the EMR probably increased the data accuracy, and to the authors' knowledge, GP consultations by patients with cancer has not been explored by this method before.

The participating GPs were comparable to Norwegian GPs regarding sex, with a geographic distribution representing both urban and rural districts.

The present study is explorative and conclusions cannot be drawn on generalisability, owing to a limited sample of responders and lack of information on response rate. Each GP had to be contacted directly by mail and participation depended on their goodwill. It could represent a selection bias if the GPs who had only a few or no patients with cancer on their list chose not to participate, or if some GPs were reluctant to participate because they did not feel comfortable using an electronic tool. On the other hand, Norwegian GPs' patient lists consist of patients who have chosen their GP, and the lists represent a mixed sample. This means that most GPs probably have patients with cancer on their lists. Therefore, there is no reason to believe that these GPs differ from the other Norwegian GPs regarding their work with patients with cancer.

Another limitation is that the accuracy of the diagnoses is unknown. GPs may have made coding mistakes, and it is also possible that a cancer diagnosis alone may have been used in cases of symptoms, side effects, or complications where the cancer is believed to be the cause. A Swedish study showed a correlation of 97% between EMR notes and diagnosis in 400 cases,^14^ whereas a Norwegian study showed a correlation between EMR notes and diagnosis of 85% in 839 consultations by 23 GPs.^15^

This current study included all patients registered with a cancer diagnosis during 1 year, and it is acknowledged that this is not a homogeneous group. Some may be recently diagnosed and under treatment, some may have been successfully treated but experience side effects, and some may be in need of palliative care. On the other hand, this 'mixed sample' is representative of a GP’s caseload, and this reality is what the authors wanted to explore in this study.

### Comparison with existing literature

This study showed that there are large differences among GPs in terms of contacts with patients with cancer. The most experienced GPs saw more patients with cancer, which may be explained by the fact that experienced GPs have older patient populations with higher cancer prevalence.^13^

The median age of the patients in the study correlates with the median age at first diagnosis of cancer in Norway, which is 69 years.^16^ The variation in the consultation rate among patients could be explained by different cancer diagnoses and different phases of the disease. Follow-up programmes differ between the cancer types regarding frequency of visits and to what extent the GP is involved in follow-up. In this study, the median number of consultations per patient was five, compared with a mean number of 2.7 GP consultations per person in Norway in 2017.^17^ Of course, it cannot be known how many patients with cancer did not consult their GP during this 1 year. However, the increased consultation rates are in line with findings of several studies regarding primary healthcare use among patients with cancer.^3,4,8–10,18^ A Dutch study comprising several cancer types found that, compared with matched controls, patients with cancer had significantly more GP consultations (3.5 versus 2.7 per year).^18^ A UK study found that patients with breast and colorectal cancer had one more GP consultation per year compared with controls, up to 5 years after diagnosis for breast cancer, and up to 9 years for colorectal cancer. Patients with prostate cancer consulted GPs up to three more times per year than controls and this trend persisted up to 15 years post-diagnosis.^9^ Other, Dutch studies found that patients with colorectal cancer had 54% more face-to-face contacts with GPs compared with reference patients in the first year after diagnosis,^8^ as well as significantly more face-to-face contacts in the second, third, and sixth year after diagnosis.^3^

Studies trying to explain the cause of the increased consultation rates among patients with cancer have shown conflicting results. Jabaaij *et al* found that patients with cancer more often had a chronic comorbid condition than their matched controls, and concluded that having a chronic condition increased healthcare use.^18^ Khan *et al* stated that a major contribution to the increased consultation rate in patients with prostate and breast cancer was the monitoring and administration of hormonal treatments.^9^ Brandenbarg *et al* found that patients with colorectal cancer consulted their GP for reasons related to anaemia, abdominal pain, constipation, micturition problems, and psychological problems related to the cancer; however, they did not find any increased healthcare use resulting from comorbid conditions.^8^ A study by Roorda *et al* found that patients with breast cancer had more face-to-face contact with their GP for reasons related to breast cancer or breast cancer treatment.^4^ The main predictor of higher consultations rates was a higher age at diagnosis, possibly owing to the presence of comorbid conditions in older women. A study from Estonia interviewing patients with cancer found that even if cancer treatment took place at oncology clinics, patients consulted their GP as well.^19^ The role of GPs was seen as taking care of other diseases, providing information about cancer and its treatment, and coordinating care. However, these patients preferred to discuss cancer-related problems with oncologists. This correlates with the present authors’ findings in a study among patients with gynaecological cancer, who also preferred to discuss cancer-related issues with the gynaecologist rather than with their GP.^20^

Systematic reviews have shown a higher prevalence of depression and anxiety after cancer treatment compared with the general population.^21,22^ Data from Statistics Norway show that 10% of the main diagnoses in GP consultations in 2017 were within this diagnostic group.^17^ This is in contrast to the present study's findings, where only 4% of the main diagnoses were within the psychological group. A possible explanation is that the cancer diagnosis could mask such diagnoses and symptoms. GPs could, by using a cancer diagnosis alone, also imply consequences of the cancer such as anxiety and depression.

### Implications for practice

A study carried out by the research group showed that GPs had experience with follow-up care for patients with cancer.^5^ However, when asked if they could take on further responsibility for formal follow-up, the GPs were hesitant owing to fears of increased workload.

This study indicates that GPs already play a substantial but informal role in cancer follow-up, and a formalisation of their responsibility would probably not imply a substantial increase in workload if good information was provided to GPs as well as patients on the follow-up programme. GPs should prepare for this responsibility, and guidelines have to be adjusted according to this reality.
